# Special Issue on Basic and Translational Research in Colorectal Cancer

**DOI:** 10.3390/ijms20123095

**Published:** 2019-06-25

**Authors:** Paola Ulivi, Emanuela Scarpi, Alessandro Passardi

**Affiliations:** Istituto Scientifico Romagnolo per lo Studio e la Cura dei Tumori (IRST) IRCCS, 47014 Meldola (FC), Italy; emanuela.scarpi@irst.emr.it (E.S.); alessandro.passardi@irst.emr.it (A.P.)

**Keywords:** colorectal cancer, biomarkers, heterogeneity, liquid biopsy

## Abstract

The present editorial aims to summarize the 17 scientific papers that have contributed to this Special Issue focusing on different aspects of basic and translational research in colorectal cancer.

Colorectal cancer (CRC) is the third most commonly diagnosed cancer in males and the second in females. The genome of colon cancer cells is altered at several sites following point mutations or changes in chromosome integrity. These mutation-associated changes affect oncogenes, tumor suppressor genes and several metastasis-related genes. The microenvironment and characteristics of the host may also play an important role in influencing cancer growth. In recent years, major advances have been made in our understanding of the molecular mechanisms governing tumor growth and metastatic dissemination, and of the role played by the tumor microenvironment. Many of the findings have been implemented into clinical practice, culminating in the development of new molecular-targeted drugs. 

Molecular alterations can be used for diagnostic [1], prognostic [2,3,4,5,6,7] and predictive [7] purposes. Furthermore, the use of bioinformatics and machine learning could help to define and validate novel “omics-based” markers with important diagnostic and prognostic implications [1]. Polymorphisms in DNA repair genes have been linked to higher sporadic colorectal cancer susceptibility, some (affecting *REV3L*, *POLQ* and *NEIL3* genes) showing an association with patient survival, indicating their potential prognostic usefulness [8]. 

Angiogenesis, inflammation and the immune system also influence cancer growth and metastasization. Low VEGFR-1 and high VEGFR-2 expression in endothelial cells have been shown to affect CRC progression, indicating the former as a potential target for therapy [2]. Tumor-associated neutrophils (TANs), in particular, the neutrophil-to-lymphocyte ratio, are well-defined predictive markers for CRC. TANs contribute to tumor invasion and angiogenesis through the production of MMP-9, VEGF and HGF in both primary and metastatic tumor sites, suggesting their potential usefulness as targeted treatment [9]. The role of TANs in CRC was also discussed in the review by Nasr et al. Inflammatory biomarkers are known to influence CRC prognosis and response to chemotherapy. Acute phase proteins, inflammatory cytokines and blood cell ratios are all associated with CRC patient outcome. Given that they can easily be determined in blood, their use in clinical practice in combination with TNM staging system could facilitate the identification of high-risk patients and those who are most likely to benefit from therapy. In addition, microRNA (miRNA) has been hypothesized as capable of stratifying CRC patients for optimal drug selection [6]. miRNAs are also thought to play an important role in the metastasizing of CRC through the regulation of Wnt/βcatenin, EGFR, TGFβ and TP53 signaling pathways, and the modulation of epithelial-mesenchymal transition, a process involved in liver metastasization. miRNAs are released into exosomes, a process thought to mediate liver metastasis through the preparation of the pre-metastatic niche [10]. Although several other biomarkers have been linked to liver metastasis, none have provided sufficiently robust results to confirm their involvement [7]. Long non-coding RNAs (lncRNAs) also play an important role in different biological processes involved in cancer. In particular, *CCAT1* and *linc-ROR* have been shown to be upregulated in tumor tissue with respect to healthy tissue, whereas *ANRIL*, *MIR155HG* and *MALAT1* are downregulated. Moreover, the ratio of *CCAT1* to *ANRIL* and *MIR155HG* in normal tissue may have prognostic implications [3]. Polycystins are key mechanosensor proteins capable of responding to external and internal mechanical forces. They are known to mediate cell-to-cell and cell-to-extracellular matrix (ECM) interactions and are associated with focal adhesion and ECM proteins that are deregulated in oncogenesis. These proteins also regulate apoptosis, differentiation, cell orientation/migration, cell cycle and tissue morphogenesis. Recent findings have linked polycystins to CRC phenotypes, suggesting their potential as novel biomarkers and targets for therapy [11]. Other prognostic factors capable of discriminating between patients with different risk of metastasization are tissue fatty acids. Notarnicola et al. reported that metastatic patients showed significantly lower levels of eicosapentanoic acid and higher levels of γ-linolenic acid, suggesting that membrane lipid may influence cellular functions, making cells more or less prone to metastasis [12]. Situ et al. reported evidence of a possible relationship between MRE11-RAD50-NBS1 (MRN) expression and CRC prognosis. The MRN complex is also a promising target for treatment.

Heterogeneity is another characteristic of many tumors, including CRC. It can influence the molecular characterization of tumor tissue, now mandatory to select candidates for treatment (in particular, anti-EGFR monoclonal antibodies) to reduce the risk of false negatives that result in a non-optimal choice of therapy. Temporal molecular heterogeneity may also occur during treatment and can influence response and prognosis [13]. Liquid biopsy could potentially overcome the problem of heterogeneity as it enables tumor material originating from different tumor sites to be analyzed. In particular, cell-free circulating DNA represents a valid option for the characterization of tumors and their monitoring during treatment [13,14]. Although several molecular biomarkers have been evaluated (in both tumor tissue and liquid biopsies) as predictors of resistance to anti-EGFR monoclonal antibodies, the road from the laboratory to the clinical has turned out to be a rather winding one [15]. 

Another important issue is tumor location. CRC is not a single type of tumor, as tumors located in the right side of the colon exhibit different molecular characteristics and histology with respect to those originating in the left side [16]. Moreover, several differences exist in the pathogenesis, molecular profile, anatomy and patient outcome between colon cancer and rectal cancer, which are no longer considered as a single entity [17].

Moving from the bench to the bedside, El Halabi et al. explored the potential benefits of the use of intravenous ascorbic acid in CRC, which resulted in a shutdown of the downstream KRAS pathway in in vitro studies. In a clinical setting, this treatment appears to induce tumor regression, in addition to showing a better safety profile than that of standard treatment.

Although substantial progress has been made in relation to this type of cancer, there is still much to understand and further studies are needed to find novel and more effective treatment strategies for patient management.
